# Male and Female Infertility in the Quest for Research Support and Public Health Interest: Analysis of Two Large Databases

**DOI:** 10.7759/cureus.91335

**Published:** 2025-08-31

**Authors:** Shane A Tinsley, Jack Considine, Simisolaoluwa Olabode, John Nzasi, Keshav Lalchandani, Keavash Assani, Ali A Dabaja, Amarnath Rambhatla, Lane Shish, Logan C Hubbard

**Affiliations:** 1 Vattikuti Urology Institute, Henry Ford Health, Detroit, USA; 2 Urology, Wayne State University, Detroit, USA; 3 Urology, Michigan State University College of Human Medicine, East Lansing, USA; 4 Urology, Vanderbilt University Medical Center, Nashville, USA; 5 Urology, Wright State University Boonshoft School of Medicine, Dayton, USA; 6 Urology, University of Minnesota, Minneapolis, USA

**Keywords:** female infertility, grants, health awareness, male infertility, public health

## Abstract

Objective

The rate of infertility is increasing and affects males and females relatively equally. With the rise of infertility cases impacting both males and females, there will be an increased need for screening, diagnostic, and treatment modalities to meet the growing demand. However, it is unclear if disparities exist regarding research funding allocation and public interest. The purpose of this study is to compare research expenditure and public interest between male and female infertility.

Methods

The National Institutes of Health (NIH) Research Portfolio Online Reporting Tools Expenditures and Results (RePORTER; duration: 1985-2022) and Google Trends (duration: 2004-2023) United States datasets were used to assess research support and public interest, respectively. Data were obtained from the NIH RePORTER, including grants identified by validated MeSH terms and verified with an in-depth review of the project title, abstract, aims, and public health relevance. Terms were searched separately and simultaneously in Google Trends, which provided relative search volume. To assess inferential statistics, Mann-Whitney U and chi-square tests were performed. Regarding Google Trends, the Wilcoxon test was used to assess changes in the relative search volume over time.

Results

A total of 589 grants were funded. In aggregate, female infertility had more research funding in total ($100,931,789 vs. $71,684,515). Moreover, female infertility had more funding per grant ($315,192 vs. $286,371; p < 0.0001) compared to male infertility. In Google Trends, the terms (separately) decreased by -38 points (p < 0.0001) for male infertility and -15 points (p = 0.9) for female infertility. When analyzed separately (median: 36 for male infertility vs. 34 for female infertility) and simultaneously (median: 35 for male infertility vs. 14.5 for female infertility), the term "male infertility" had significantly higher relative search volume than "female infertility" (p < 0.0001).

Conclusion

Disparities exist in research support and public interest in infertility, with more research support toward female infertility and public interest toward male infertility. Moreover, there was a significant decrease in public interest in male infertility, which could contribute to decreased awareness that could delay diagnosis and treatment. Further research should be devoted to identifying causes for the disparities in research funding allocation and public health interest observed in our study.

## Introduction

Infertility is defined as the inability to achieve pregnancy after at least 12 months of regular and unprotected vaginal intercourse [1-4]. The incidence and prevalence of infertility have 0been increasing in the Western world in recent years [5]. The National Institutes of Health (NIH) is a major contributor to researchers and scientists within the United States of America (USA) [6], though the process of procuring a research grant has become increasingly difficult [7]. Additionally, previous studies have alluded to disparities in the allocation of research funding into specific research topics [8,9]. For instance, among 509 grants awarded to urology departments in the USA between 2010 and 2019, 207 (56.4%) of grants were investigating urologic oncologic diseases alone in contrast to other genitourinary topics [8]. Hence, it is important to assess the allocation of research funding toward essential healthcare topics to ensure equitable distribution of research funding, given that the prevalence of male-to-female infertility is roughly equivalent [10,11].

Furthermore, patients are increasingly turning to online search engines to gain knowledge about various healthcare topics, such as risk factors, screening, diagnosis, and treatment of various diseases and conditions [12]. Search trends related to public health topics could illustrate perceived importance among patients regarding awareness, a desire for health optimization, or treatment of potential conditions. For instance, large proportions of the general population use Google to obtain medical information, and prior studies have utilized this search engine to assess public health interest in various topics, including within urology [13-16]. Our team utilized Google Trends since online search inquiries could reflect an interest or awareness in health topics. Thus, it is important to devote resources to investigating trends and discrepancies in public health interest in various health awareness topics.

Specifically, we examine differences in NIH research funding and public search interest between male and female infertility. We hypothesized that research expenditure and public health interest in male and female infertility would increase over time; however, male infertility would receive less research funding and public interest compared to female infertility. This study aims to compare research funding and public interest in male versus female infertility in the United States. 

Of note, this project was presented as an abstract at the American Society of Reproductive Medicine (ASRM) meeting on 10/1/2023.

## Materials and methods

Databases

The NIH Research Portfolio Online Reporting Tools Expenditures and Results (RePORTER) database is a publicly available dataset that discloses all research expenditures from the NIH from 1985 to the present [17]. Information such as fiscal reports, grantee characteristics, publications, and news/media coverage is openly accessible through the dataset.

The software algorithm and database, Google Trends, were utilized to examine public health interest. Google Trends provides measures of the relative search volume (RSV), a relative value for the number of times a term has been input into Google [18]. The RSV score is calculated in a range from 0 to 100: a score of 0 equates to the lowest volume of RSV, and 100 corresponds to the highest RSV for each term during the designated period. The RSV is outputted based on designated criteria inputted into Google Trends, including time duration, geographic location, and search term(s). One or more search terms can be input into the software application at a time to identify and assess changes in RSV. Data are available from January 1, 2004, to the present. Furthermore, data can be stratified by geographic region (i.e., states in the USA). For this study, the RSV was used as our surrogate for public health interest [19].

Covariates

Covariates related to the topic of the research project, research technique(s), fiscal information, and grantee characteristics were obtained from NIH RePORTER. The RSV was extracted from the Google Trends dataset.

Data collection 

Inclusion criteria for the NIH RePORTER dataset included the following: (1) projects between 1985 and 2022; (2) grantees within the USA; (3) grant topics related to the topics of male infertility or female infertility - exclusively using validated MeSH terms (Appendix 1); and (4) projects with activity codes dedicated to research purpose grants - as defined by the NIH (Appendix 2). MeSH terms were identified using the NIH MeSH term search engine. General terms related to the diagnosis of male and female infertility were identified first. Thereafter, additional MeSH terms were identified from MeSH term hierarchical categorical trees. Our team solely included research purpose grants to capture projects dedicated to the development of knowledge and technologies related to infertility. The exclusion criteria for our project were as follows: projects in 2023 or 2024 and projects funded using activity codes outside our list. Projects that did not have activity codes defined as "research purpose grants by the NIH were also excluded.

Funding awards were further queried in relation to male or female infertility by six reviewers. Members of our research team reviewed (1) the title of the grant, (2) the abstract of the grant, (3) the aims of the grant, and (4) the public health relevance of the grant. Projects were included for final analysis if at least one of the following metrics aligned solely with either female infertility or male infertility. If none of those metrics aligned with either female infertility or male infertility, they were excluded from data analysis. Moreover, grants that were deemed to be associated with both female infertility and male infertility were excluded as well. The rationale was our team's inability to ascertain the extent the project investigated female infertility and male infertility within the project. If there was discordance as to whether research was more closely related to male or female infertility, it was addressed via independent review by physicians with infertility sub-specialty training until a consensus was reached.

MeSH terms initially populated 546 projects that were designated with female infertility, and 412 projects were designated with male infertility. Upon review of the grants, among projects initially designated with female infertility, 304 were found to be associated with female infertility, and 12 projects were deemed to be associated with male infertility. The remaining funded grants, n = 230, were excluded as they did not meet our inclusion criteria. For the grants populated from the "male infertility" MeSH terms, 264 and three projects were deemed to be associated with "male infertility" and "female infertility" terms, respectively. Our team also searched all "male infertility" and "female infertility" terms together to identify any missed grants, which populated 17 unique grants and yielded only six male infertility projects. In our review, there were 13 grants that were sent for additional review by physicians with infertility sub-speciality training. Of those cases, 10 were deemed to be aligned with male infertility, one was aligned with female infertility, and two were not assigned either male infertility or female infertility.

Information not explicitly available in NIH RePORTER, specifically the educational attainment of principal investigator (PI) (i.e., MD, PhD), was collected from an online search, utilizing (1) designated institution and (2) email address available in NIH RePORTER. Information that could not be obtained either through the NIH RePORTER database or online search was defined as missing for the purpose of our project.

For the Google Trends portion of this study, we focused on data collection between January 1, 2004, to December 27, 2023, in the USA (51 regions). Our team did not utilize categories or filters for the purpose of querying search terms in the software application. The reason was not to accidentally exclude any uncategorized or unfiltered searches from those topics. Information outside of our desired time duration or geographic area was excluded from data collection and analysis. The following search terms were used within the Google Trends software application: "male infertility" and "female infertility". The search terms were input as "male infertility" (solely), "female infertility" (solely), and "male infertility" + "female infertility" (simultaneously).

Endpoints 

The main endpoints in the NIH RePORTER dataset were finding the amount of funding (per grant/overall), grant awards, peer-reviewed publications per award, documented NIH-clinical studies per award, new/media events per award, and research technique per award between male infertility and female infertility. The main endpoint in the Google Trends dataset was finding the RSV for male infertility versus female infertility.

Data analysis

All continuous and categorical variables were reported as medians with interquartile ranges (IQR) and proportions with percentages (%), respectively. Inferential statistics were obtained by performing the Mann-Whitney U test and Pearson's chi-square test on continuous and categorical covariates to compare male and female infertility, respectively. To assess changes in RSV scores over time in our Google Trends dataset, we used the Wilcoxon test to compare RSV scores between the years 2004 and 2023. The Wilcoxon test was also used to compare changes in the number of funded research projects in the NIH RePORTER dataset between the first and last five years for male and female infertility. All statistical analyses were performed using RStudio (RStudio Team, Boston, MA) and Microsoft Excel (Microsoft® Corp., Redmond, WA), and all tests were two-sided with a statistical significance defined as p-value < 0.05.

Internal review board exemption

This study was exempt from Internal Review Board (IRB) approval as the pertinent data were publicly available.

## Results

NIH RePORTER data

A total of 589 awards were identified from the NIH RePORTER database. Of those funding awards, 282 were designated to solely investigate male infertility, and 307 were designated to solely investigate female infertility. Most grant awards were R-Category Awards (i.e., R00, R01, R21), awards for research projects with high likelihood of scientific merit, for both male infertility (n = 254) and female infertility (n = 288). The National Institute of Child Health and Human Development (NICHD) was the most common research funding administrator for male infertility (n = 194) and female infertility (n = 202). Most principal investigators did not hold an MD/DO degree (i.e., PhD, ScD, or DVM) for male infertility (n = 60) and female infertility (n = 57). Funded male infertility research was conducted at nine institutions versus 62 institutions researching female infertility. Further information regarding the characteristics of the covariates included in our analysis is presented in Table 1.

**Table 1:** Descriptive Statistics of Research Support Toward Male and Female Infertility From the National Institutes of Health in the United States Between 1985 and 2022 This table compares the amount of funding, supported awards, peer-reviewed publications, clinical studies, and news/media coverage received for National Institutes of Health-funded projects pertaining to the topics of male or female infertility in the USA between 1985 and 2022. Statistical significance was defined as p-value < 0.05. All statistically significant p-values are bolded. R-Category Awards = R00, R01, R03, R15, R16, R21, R33, R34, R35, R36, R37, R50, R56, R61; P-Category Awards = P01, P42; U-Category Awards = U01, U34 Interquartile Range = IQR; National Institutes of Health = NIH; Doctor of Medicine = MD; Doctor of Osteopathic Medicine = DO; Doctor of Philosophy = PhD; Doctor of Science = ScD; Doctor of Veterinary Medicine = DVM

Category	Male Infertility	Female Infertility	p-value
Median Funding per Award	$286,371 (IQR: 161,840-321,724)	$315,192 (IQR: 235,831-389,428)	<0.0001
Total Funding Awarded	$71,684,515	$100,931,789	-
Median Awards per Year	7 (IQR: 4-10)	7 (IQR: 1-10.75)	0.4404
Total Awards	282	307	-
Median Publications Per Award	14	9	<0.001
Median Documented NIH Clinical Studies Per Award	0	0	0.946
Median News/Media Events Per Award	0	0	0.052
Total Research Techniques for Project Awards	282	307	<0.0001
Basic Science	102	47	
Translational Science	161	226	
Clinical Science	19	34	
Total of Activity Codes for Project Awards	282	307	<0.001
R-Category Awards	254	288	
P-Category Awards	24	6	
U-Category Awards	4	13	
Total National Institutes of Health Conferring Institutes	282	307	<0.0001
National Cancer Institute	5	3	
National Human Genome Research Institute	1	0	
National Heart, Lung, and Blood Institute	0	8	
National Institute on Aging	1	8	
National Institute of Biomedical Imaging and Bioengineering	0	4	
National Institute of Child Health and Human Development	194	202	
National Institute on Drug Abuse	0	1	
National Institute of Diabetes and Digestive and Kidney Diseases	5	22	
National Institute of Environmental Health Sciences	13	33	
National Institute of General Medical Sciences	56	26	
National Institute for Occupational Safety and Health	4	0	
Office of the Director	3	0	
Educational Background of Principal Investigators	75	80	0.052
MD/DO Training	8	20	
Non-MD/DO Training (i.e., PhD, ScD, or DVM)	60	57	
Missing Educational Training	6	3	
Number of Funded Institutions	9	62	-

**Figure 1:**
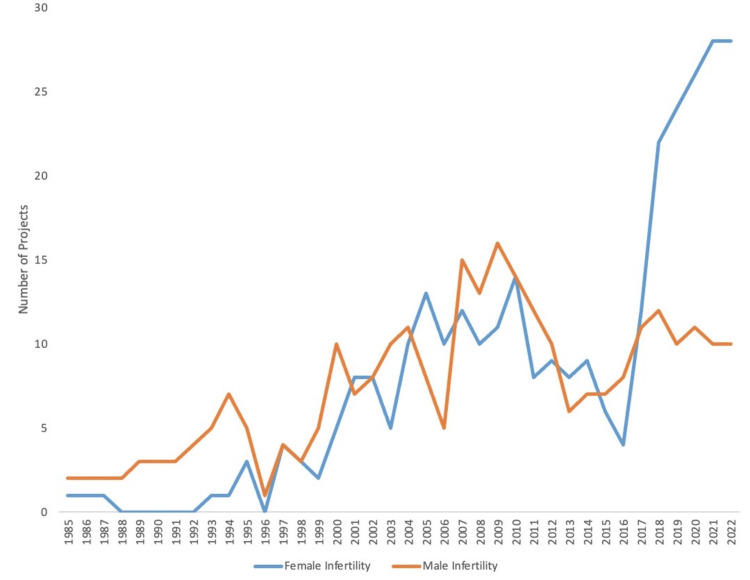
Number of Grants Funded Pertaining to Male Infertility and Female Infertility per Year From 1985 to 2022 The number of funding awards per year from the National Institutes of Health towards projects investigating male infertility or female infertility in the USA between 1985 and 2022.

Overall, the number of research projects investigating male and female infertility in the USA has increased between 1985 and 2022. The number of grants investigating male infertility increased from two in 1985 to 10 in 2022, while the number of grants increased from two in 1985 to 28 in 2022 for female infertility. Both male infertility and female infertility were found to have a significant increase in the number of funded research projects between the first and last five years in the study period (both p < 0.0001). Of note, male and female infertility had a median of seven funded research projects per year (p = 0.052). Further information can be found in Figure 1.

In our analysis of projects focusing on male versus female infertility, projects focused on male infertility were found to have a lower amount of funding per grant ($286,371 vs. $315,192; p < 0.0001), but more peer-reviewed publications per grant (14 vs. 9; p < 0.0001). Projects focusing on male or female infertility were found to have a similar number of NIH-documented clinical studies (0 vs. 0; p = 0.95) and news/media coverage (0 vs. 0; p = 0.052) per grant. However, projects focused on male infertility had less total funding ($72,715,311 vs. $101,188,793), clinical sciences-oriented awards (19 vs. 34), and translational science-oriented awards (161 vs. 226). Male infertility grants were found to have more basic science-oriented awards (102 vs. 41), as summarized in Table 1. 

Google Trends data

The search terms (separately) had overall medians of 36 (IQR: 33-52.25) for male infertility and 34 (IQR: 28.75-40) for female infertility (p-value < 0.0001). When searched simultaneously, the search terms "male infertility" and "female infertility" had median overall RSV of 36 (IQR: 33-46) and 13.5 (11-16), respectively (p-value < 0.0001). The search terms (separately and simultaneously) were searched in 35 regions in the USA for male infertility and 21 regions for female infertility (p-value < 0.01). Further information can be located in Table 2.

**Table 2:** Geographic Regions in the United States and Their Associated Relative Search Volume (Public Health Interest) Into Male and Female Infertility Between 2004 and 2022 The relative search volume (ranging from 0 to 100) per region (n = 51) in the USA for the search terms "male infertility" and "female infertility" searched separate and simultaneously between the years 2004 and 2023.

Region	Male Infertility (Separately)	Female Infertility (Separately)	Male Infertility (Simultaneously)	Female Infertility (Simultaneously)
Alabama	88	0	100	0
Alaska	0	0	0	0
Arizona	68	74	69	31
Arkansas	0	0	0	0
California	75	69	72	28
Colorado	66	64	72	28
Connecticut	86	0	100	0
Delaware	0	0	0	0
District of Columbia	0	0	0	0
Florida	70	73	70	30
Georgia	76	75	71	29
Hawaii	0	0	0	0
Idaho	0	0	0	0
Illinois	78	76	71	29
Indiana	88	100	68	32
Iowa	0	0	0	0
Kansas	94	0	100	0
Kentucky	90	0	100	0
Louisiana	83	0	100	0
Maine	0	0	0	0
Maryland	92	88	72	28
Massachusetts	83	69	75	25
Michigan	84	81	72	28
Minnesota	94	0	100	0
Mississippi	96	0	100	0
Missouri	91	84	72	28
Montana	0	0	0	0
Nebraska	78	0	100	0
Nevada	74	0	100	0
New Hampshire	85	0	100	0
New Jersey	87	87	71	29
New Mexico	0	0	0	0
New York	100	83	74	26
North Carolina	78	72	73	27
North Dakota	0	0	0	0
Ohio	87	81	72	28
Oklahoma	82	0	100	0
Oregon	69	69	71	29
Pennsylvania	78	81	70	30
Rhode Island	0	0	0	0
South Carolina	79	93	67	33
South Dakota	0	0	0	0
Tennessee	80	0	100	0
Texas	83	65	76	24
Utah	100	0	100	0
Vermont	0	0	0	0
Virginia	75	72	72	28
Washington	73	72	71	29
West Virginia	0	0	0	0
Wisconsin	75	0	100	0
Wyoming	0	0	0	0

**Figure 2:**
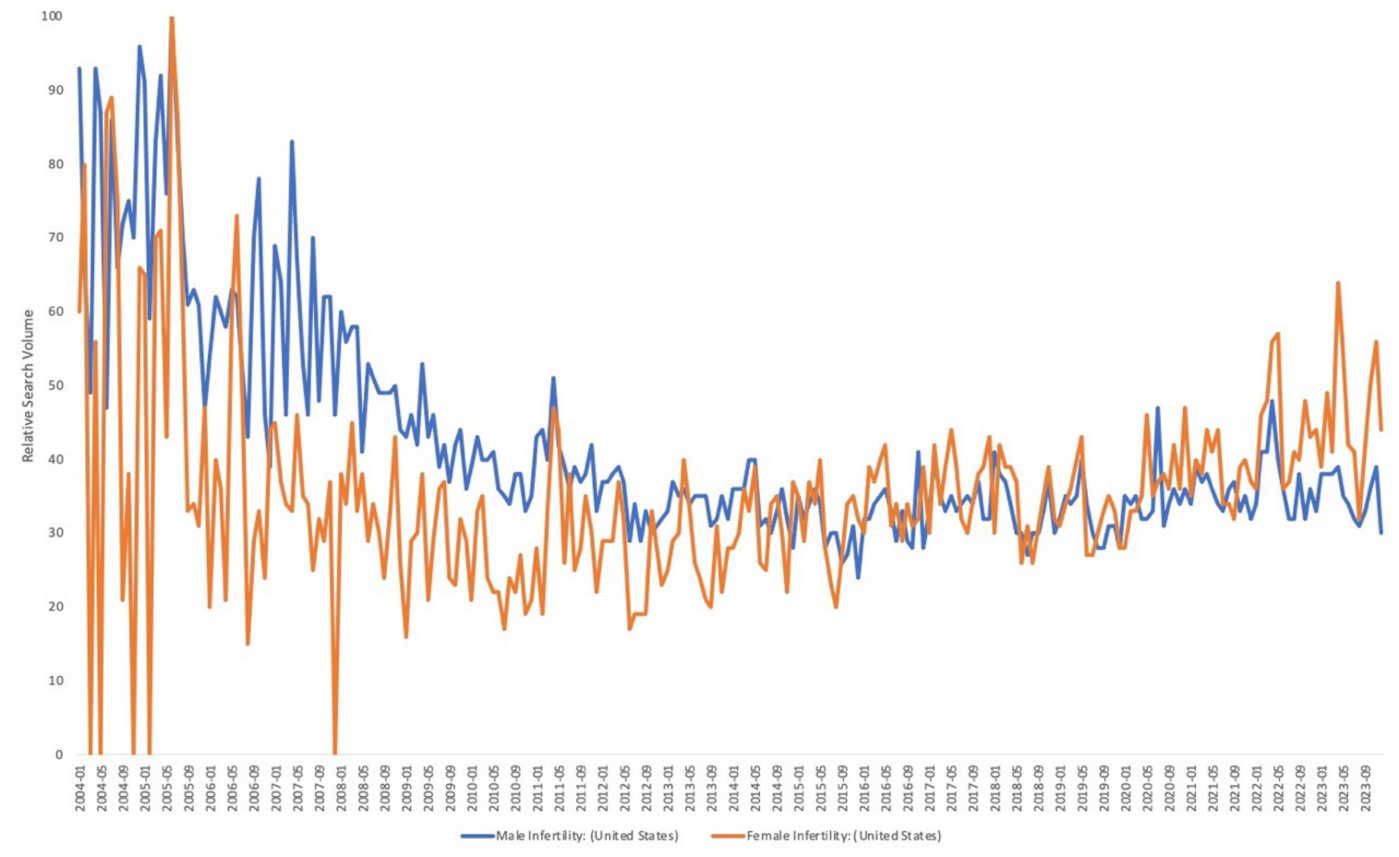
Relative Search Volume (Public Health Interest) Into Male and Female Infertility Terms Between 2004 and 2022 in the United States (Searched Separately) The relative search volume (ranging from 0 to 100) into the search terms "male infertility" and "female infertility" searched separately from each other in the USA between 2004 and 2023.

**Figure 3:**
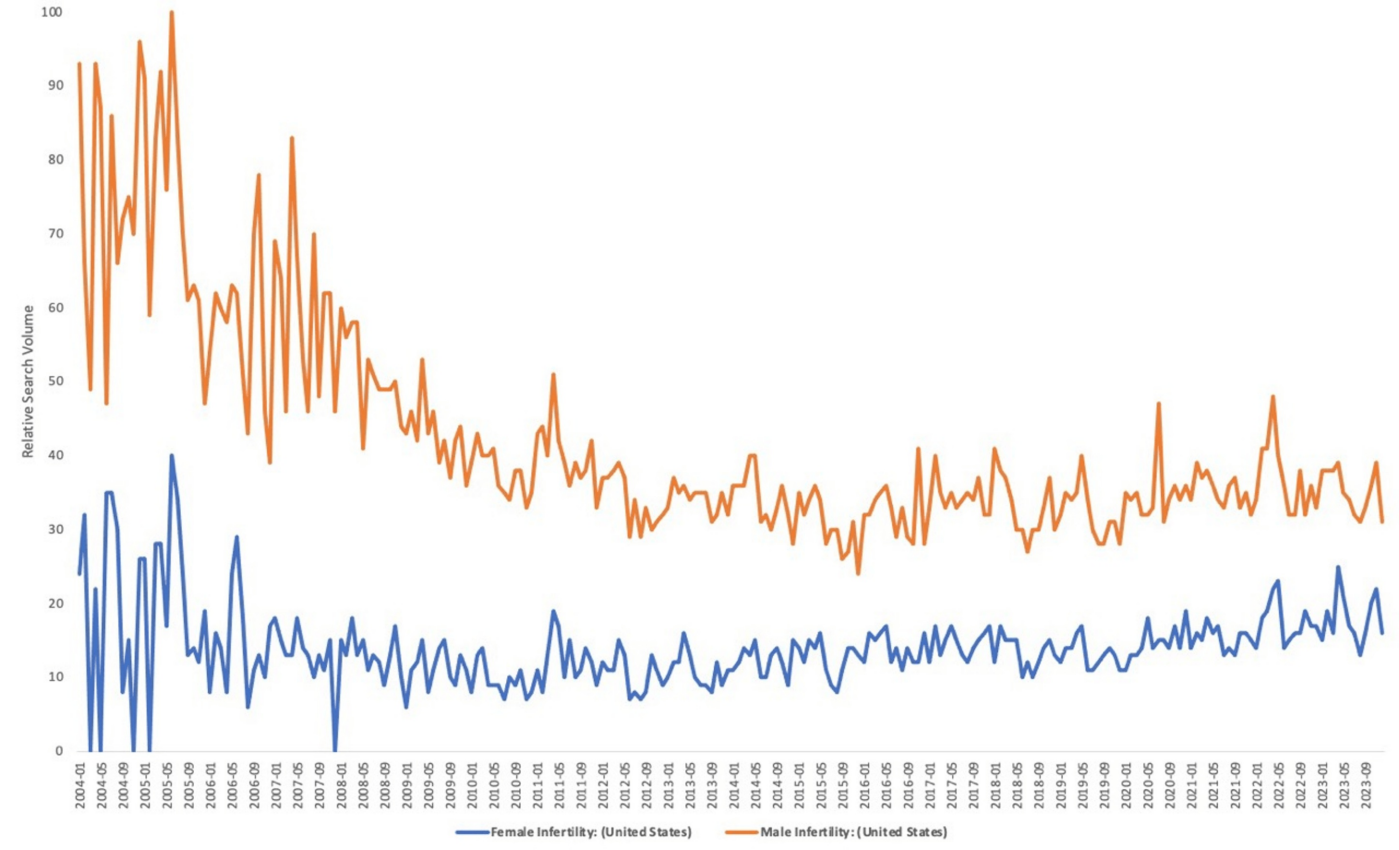
Relative Search Volume (Public Health Interest) Into Male and Female Infertility Terms Between 2004 and 2022 in the United States (Searched Simultaneously) The relative search volume (ranging from 0 to 100) into the search terms "male infertility" and "female infertility" searched simultaneously with each other in the USA between 2004 and 2023.

The median RSV decreased between the years 2004 and 2023 for each search term: (73.5-35.5; p < 0.0001) for male infertility and (58-43; p = 0.9) for female infertility. This was confirmed with male infertility and female infertility (simultaneously), which indicated an RSV for male infertility significantly decreased from a median of 73.5 to 35.5 (p < 0.0001), while female infertility decreased slightly from a median of 23 to 16 (p = 0.8). Further information is located in Figures 2-3.

## Discussion

To the best of our knowledge, this represents the first study examining research funding allocation and public health interest regarding male infertility and female infertility in the USA. Many of the results from our study are noteworthy, specifically the disparities in research support/subsequent research outcomes and public health interest between male and female infertility. Overall, a total of 589 grants were funded, with female infertility receiving more research funding in total ($100,931,789 vs. $71,684,515) and funding per grant ($315,192 vs. $286,371; p < 0.0001) compared to male infertility. However, in terms of public interest, male infertility had higher RSV scores separately (median: 36 for male infertility vs. 34 for female infertility) and simultaneously (median: 35 for male infertility vs. 14.5 for female infertility) compared to female infertility (p < 0.0001).

Collectively, the data achieved from the NIH RePORTER database met our initial expectations. It was unsurprising to see that R-Category Awards (i.e., R00, R01, and R21) were the most common grant awards (90% for male and 94% for female infertility). Moreover, the NICHD and principal investigators without an MD/DO degree were the most common funding administrators (69% for male and 66% for female infertility) and funded investigators (80% for male and 71.25% for female infertility), respectively. The results pertaining to the R-Category Awards covariate were unsurprising because the most administered grant from the NIH is an R01, which fell under the R-Award category in our project [20]. Second, the NICHD serving as the most common administrator was not unexpected due to their disclosed research priorities in human development (including fertility/infertility) [21]. Finally, due to the shortage of physician scientists in the USA, we did not expect to see a large proportion of investigators with an MD/DO degree [22].

Upon examination of the NIH RePORTER data, it was striking to observe that only nine institutions conducted NIH-funded male infertility research, while 62 institutions conducted NIH-funded female infertility research. It can be presumed that, due to historical biases or healthcare engagement among individuals struggling with infertility, female factor infertility would garner a larger proportion of research expenditure [23-25]. However, it was surprising to observe that research interest in male factor infertility is scarce among academic and research institutions across the USA. It is plausible that this observation stems from the limited distribution of fellowship-trained urologists in men's health/male infertility (andrologist) at academic and/or research institutions. Since current evidence suggests that there is a disparity in the number of trained andrologists in the USA compared to the male population, they seek to serve [26]. Thus, this leads to a smaller population of male infertility experts conducting research in this area. However, the exact reasons for this observation are unknown but should be an area of future investigation.

In terms of our examined endpoints using the NIH RePORTER data, it was postulated that research expenditure into both male and female infertility would increase over time, stemming from the increasing incidence and prevalence of infertility cases, despite the decrease in the NIH budget in recent years [5,7]. However, historically, a couple’s inability to conceive was attributed to women contributing to potentially poor research support into male factor infertility [23,24]. There is still much to learn about the biology of male infertility (i.e., impact of DNA, RNA, and centrioles), but research support is stifled due to current infertility research priorities favoring female infertility [24]. It was therefore unsurprising to observe that male infertility had a lower amount of total grants and funding (per grant/overall).

However, it was surprising to see the uptick in research expenditure into female infertility in recent years (the last five years), in contrast to our initial expectation that female infertility research would have garnered more grants throughout the entire study duration. This may be attributed to increased advocacy and awareness toward female reproductive health (i.e., Women’s Health Movement), which in turn influenced the landscape of research interests, or alternatively stemming from shifting demands in healthcare and/or the utilization of healthcare habits between males and females [27].

This is especially true given that female patients are much more likely to participate in and receive healthcare services in contrast to their male counterparts [25]. For instance, in a cross-sectional study of 15,162 participants, of whom 10.1% of men and 12.5% of women disclosed they had infertility issues, 57.3% (95% CI: 53.6-61.0) of women sought help compared to 53.2% (95% CI: 48.1-58.1) of men [25]. Those findings, in conjunction with our analysis, imply that demands for healthcare services related to infertility are higher for the female patient population, which in turn could influence the research landscape due to the supply-and-demand model for novel therapies.

Conversely, the finding that male infertility grants produced more peer-reviewed publications per grant was interesting, given that one would assume less research support would contribute to fewer research outputs. One possible explanation for this observation is the production of additional peer-reviewed editorials, commentaries, and reviews compared to the research landscape in female infertility. Because the demands (i.e., resources and time) are lower to produce editorials and commentaries, it is possible that facilitated the increased number of peer-reviewed publications in contrast to purely original scientific investigations being associated with funded research projects. Additionally, it is possible that a larger proportion of research projects in male infertility research could be the result of the unique circumstances contributing to finer incremental scientific investigation compared to female infertility. More specifically, basic science research is more incremental, producing more peer-reviewed publications compared to other research techniques. In turn, the large proportion of basic science projects related to male factor infertility likely led to our observations. However, there is no strong evidence to support these claims at this time, and our team recommends further research into what factors contribute to varying degrees of research productivity in research-funded grants.

Additionally, it was unexpected that male and female infertility research garnered a similar number of news/media events per award. Initially, we thought female infertility would collect more news/media events compared to male infertility. This was based on our assumption that historical perceptions and biases of infertility disproportionately affect women and higher healthcare engagement among female patients with infertility [23-25]. The reason for this phenomenon is unknown at this time. However, the limited media engagement regarding scientific investigations and advancements in male and female infertility should be a topic of future discussion, because the dissemination of ongoing and future research into infertility is a great avenue to increase awareness and health/scientific literacy regarding infertility.

Contrary to our initial belief, results from our Google Trends analysis showed that public health interest in male and female infertility has been decreasing over time. We observed that male infertility decreased by 38 RSV (p < 0.0001), while female infertility decreased by 15 RSV (p = 0.9). With larger proportions of the population struggling with their fertility, an increase in search queries related to those topics proportional to the prevalence of infertility would be logical and expected [5]. The decline in public health interest in infertility topics could be explained by shifting preferences and ideals within the general population, specifically a greater number of the general population being eager to delay or forgo having children for reasons such as higher education or advanced employment [28].

Additionally, it was unexpected to see that male infertility (median RSV: 36) garnered more public health interest over time, compared to female infertility (median RSV: 13.5) when searched simultaneously (p < 0.0001). This could stem from increased awareness and advocacy from organizations toward educating the public about the burden male infertility poses on the general population in comparison to female infertility [29,30]. However, it could also stem from inherent limitations associated with utilizing data that are relative in nature (i.e., scores are generated based on time points with the highest number of absolute searches), which could have been different between the search terms "male infertility" and "female infertility" [19]. However, this is unlikely since searches were completed separately and simultaneously to account for potential differences in the total absolute search volume into each respective topic.

Though many of the findings in our project are noteworthy, our study is not without its limitations. First, this project is observational in nature and is confined by the limitations of our research techniques (i.e., manual review and interpretation of each funding award). Second, our project utilized MeSH terms to identify projects related to infertility, which could have missed research that did not use specified MeSH terms in the description of their objective(s). However, MeSH terms, highly organized and continuously managed sets of scientific terms by the NIH to increase scientific communication and categorization of information, are a very accurate representation of the project's aims. Thus, it is unlikely that a significant number of projects were missed, if any. Moreover, a limitation we encountered with the NIH RePORTER database was that grants are continuously being awarded and recorded into the database, which, depending on when the data were accessed, could have influenced our analysis and results. Thus, the rationale for the exclusion criteria for the years 2023 and 2024 was to obtain accurate measurements of projects related to male or female infertility in each calendar year, due to some grants still being awarded/processed. Finally, in our analysis of NIH RePORTER data, we did not perform an inter-rater readability analysis for the designation of different research projects. Regarding Google Trends, their software does not provide absolute numbers nor granularity pertaining to the characteristics of those making the searches for specific topics or phrases, which could have been beneficial in the interpretation of our results. Moreover, we were unable to directly ascertain public interest using Google Trends, so we used their RSV scores as a proxy for public health interest in a retrospective analysis. Finally, missing data, specifically terminal degree(s), were obtained from online searches, which could be vulnerable to inaccuracy. However, our team attempted to mitigate this limitation by using professional websites (e.g., institutional websites) and documents (e.g., resume and/or curriculum vitae) to obtain and verify information. Of note, our observations in the NIH RePORTER and Google Trends databases might not be generalizable internationally.

## Conclusions

The rates of infertility are increasing in the USA, and research expenditure and public health awareness can contribute to more favorable outcomes in the diagnosis and treatment of infertile couples. This study showed disparities in male and female infertility regarding research support/subsequent research outcomes, as well as public health interest. These findings call for a shift in health research policy, which should aim at providing an equitable distribution of research funding for public health concerns. Moreover, efforts should aim to investigate the reason for these observed findings and determine the best strategies to promote equitable resource allocation and public interest in both male and female fertility topics. Future efforts should aim to incorporate qualitative research methodology, such as focus groups, to learn from grant administrators and patients/general population to understand factors contributing to our observations.

## Appendices

Appendix 1 - MeSH terms

Search for Male Infertility (Excluding Female Infertility)

“Male Infertility” OR “Sterility, Male” OR “Male Sterility” OR “Subfertility, Male” OR “Male Subfertility” OR “Sub-Fertility, Male” OR “Male Sub-Fertility” OR “Sub Fertility, Male” OR “Aspermia” OR “Asthenoteratozoospermia” OR “Asthenoteratozoospermias” OR “Astheno Teratozoospermia” OR “Astheno Teratozoospermias” OR “Teratozoospermia, Astheno” OR “Teratozoospermias, Astheno” OR “Hypospermatogenesis” OR “Hypospermatogeneses” OR “Low Sperm Count” OR “Low Sperm Counts” OR “Sperm Count, Low” OR “Sperm Counts, Low” OR “Oligoasthenoteratozoospermia” OR “Oligoasthenoteratozoospermias” OR “Oligozoospermia” OR “Cryptozoospermia” OR “Cryptozoospermias” OR “Cryptospermia” OR “Cryptospermias” OR “Sertoli Cell Only Syndrome” OR “Del Castillo Syndrome” OR “Germinal Cell Aplasia” OR “Teratozoospermias” OR “Abnormal Spermatozoa” OR “Abnormal Spermatozoas” OR “Spermatozoa, Abnormal” OR “Spermatozoas, Abnormal” OR “Teratospermia” OR “Teratospermias” OR “Globozoospermia” OR “Globozoospermias” NOT “Female Infertility” NOT “Sterility, Postpartum” NOT “Postpartum Sterility” NOT “Subfertility, Female” NOT “Female Subfertility” NOT “Sub-Fertility, Female” NOT “Female Sub-Fertility” NOT “Sub Fertility, Female” NOT “Sterility, Female” NOT “Female Sterility”

Search for Female Infertility (Excluding Male Infertility)

“Female Infertility” OR “Sterility, Postpartum” OR “Postpartum Sterility” OR “Subfertility, Female” OR “Female Subfertility” OR “Sub-Fertility, Female” OR “Female Sub-Fertility” OR “Sub Fertility, Female” OR “Sterility, Female” OR “Female Sterility” NOT “Male Infertility” NOT “Sterility, Male” NOT “Male Sterility” NOT “Subfertility, Male” NOT “Male Subfertility” NOT “Sub-Fertility, Male” NOT “Male Sub-Fertility” NOT “Sub Fertility, Male” NOT “Aspermia” NOT “Asthenoteratozoospermia” NOT “Asthenoteratozoospermias” NOT “Astheno Teratozoospermia” NOT “Astheno Teratozoospermias” NOT “Teratozoospermia, Astheno” NOT “Teratozoospermias, Astheno” NOT “Hypospermatogenesis” NOT “Hypospermatogeneses” NOT “Low Sperm Count” NOT “Low Sperm Counts” NOT “Sperm Count, Low” NOT “Sperm Counts, Low” NOT “Oligoasthenoteratozoospermia” NOT “Oligoasthenoteratozoospermias” NOT “Oligozoospermia” NOT “Cryptozoospermia” NOT “Cryptozoospermias” NOT “Cryptospermia” NOT “Cryptospermias” NOT “Sertoli Cell Only Syndrome” NOT “Del Castillo Syndrome” NOT “Germinal Cell Aplasia” NOT “Teratozoospermias” NOT “Abnormal Spermatozoa” NOT “Abnormal Spermatozoas” NOT “Spermatozoa, Abnormal” NOT “Spermatozoas, Abnormal” NOT “Teratospermia” NOT “Teratospermias” NOT “Globozoospermia” NOT “Globozoospermias”

Appendix 2 - NIH research grant activity codes

Codes for NIH Grants (Solely Research Purpose Grants)

R00, R01, R03, R15, R16, R21, R33, R34, R35, R36, R37, R50, R56, R61, RC1, RC2, RC3, RC4, RF1, RL1, RL2, RL9, P01, P42, PM1, PN1, RM1, UA5, UC1, UC2, UC3, UC4, UC7, UF1, UG3, UH2, UH3, UH5, UM1, UM2, U01, U19, U34, U3R, DP1, DP2, DP3, DP4, DP5.
